# Possibilities and Challenges of Delivering Health-Related Small Group Interventions Online: Scoping Review

**DOI:** 10.2196/43783

**Published:** 2023-06-20

**Authors:** Katharina Preuhs, Mariska Klein Velderman, Pepijn van Empelen

**Affiliations:** 1 Netherlands Organization for Applied Scientific Research Expertise Group Child Health Leiden Netherlands

**Keywords:** small groups, online groups, group intervention, group cohesion, therapeutic alliance, scoping review

## Abstract

**Background:**

The outbreak of the COVID-19 pandemic required the transition of health-related face-to-face group interventions to an online setting. While it seems that group outcomes can be realized in an online setting, less is known about resulting potential challenges (and advantages) and how these can be overcome.

**Objective:**

The aim of this article is to explore what challenges and advantages may arise when providing health-related small group interventions in an online setting and how to overcome these challenges.

**Methods:**

Scopus and Google Scholar databases were searched for relevant literature. Effect studies, meta-analyses, literature reviews, theoretical frameworks, and research reports relating to synchronous, face-to-face, health-related small group interventions, online group interventions, and video teleconferencing group interventions were identified and screened. Findings relating to potential challenges and corresponding strategies are described. In addition, potential advantages of online group settings were explored. Relevant insights were gathered until saturation of results relating to the research questions was reached.

**Results:**

The literature indicated several aspects that require extra attention and preparation in the online group setting. These include the delivery of nonverbal communication and affect regulation, as well as the build-up of group cohesion and therapeutic alliance, which seem more challenging online. Yet there are strategies to overcome these challenges, such as metacommunication, collecting participant feedback, and providing guidance concerning technical accessibility. In addition, the online setting provides opportunities to reinforce group identity, such as by allowing independence and the ability to create homogeneous groups.

**Conclusions:**

While online, health-related small group interventions offer a considerable number of possibilities and benefits compared to face-to-face groups, there are also potential drawbacks to consider, which, if anticipated, can be to a great extent overcome.

## Introduction

### Background

Small group interventions are often used to promote health behavioral changes, psychological well-being, and treatment of mood disorders. These interventions may include group support or therapy in the context of addiction, HIV prevention, and lifestyle support (eg, among people at risk for chronic diseases or in psychotherapy or family therapy) [1-4]. There is good evidence of the effects of small group treatment in the context of mental problems, patient self-management, health promotion, and risk reduction behaviors (eg, [1,5]). Comparison with individual support shows that group interventions yield better results on some occasions, but generally have equivalent effects [1]. Compared to individual interventions, small groups may be more time- and cost-efficient [6]. In addition, in group interventions, members can benefit from the advantages of group processes, such as group comparison, modeling and identification, exchange between group members, practicing new skills, and social support [1,5-11]. As a result, favorable personal changes are found, such as positive psychological states, increased self-efficacy, and improved self-management [7,9,12], as well as unique group outcomes, such as group bond [9,10,13], social support [5,7,10,11,14], collective efficacy [15], and decreased feelings of isolation [7,8]. Previous research has examined which group characteristics and group processes facilitate the effectiveness of health-related small group interventions (eg, [5,11]). For optimal functioning, small group interventions generally seem to have some requirements that need to be met in order to be effective. These concern (1) group processes, referring to the way in which the group operates and exchanges information, and (2) characteristics of the group, referring to the features of the group and group members.

### Group Process

For effective group processes, the literature shows the importance of group cohesion and therapeutic alliance. Group cohesion has been found to be an essential condition that contributes to the effectiveness of group outcomes [11,16-18]. Group cohesion refers to group alliance, climate, and the relationships between group members and between group members and the group moderator (ie, therapeutic alliance; see below). A cohesive group contributes to feelings of belongingness and identification [5,7,13,19], trust [10,20,21], personal empowerment, and perceived social support [12]. Correspondingly, programs that establish trust have been shown to have higher retention rates than programs that do not [20,22].

In addition, processes such as self-disclosure of group members and feedback may facilitate changes and behavioral changes. Self-disclosure of group members may encourage the provision and reception of valuable feedback while requesting social validation [5] and strengthening cooperation [11]. Feedback, which can be defined as a reaction to a certain behavior to alter the future execution of that behavior, is often deployed as an intervention itself [23]. Giving and receiving feedback may therefore stimulate personal change through interpersonal influence, as it may enable various psychological effects, such as reinforcement, self-disclosure, reassurance, and affirmation [5,23].

The alliance between the group moderator and group members has also been shown to positively affect the outcomes of small group interventions, although the relationship is more minor than in the context of individual interventions [16]. Therapeutic alliance or working alliance refers to the mutual agreement between group members and the moderator regarding goals, tasks, and the extent to which there is an emotional bond between the moderator and group members. Therapeutic alliance has been shown to have an effect on therapy outcomes, regardless of type of intervention or therapeutic approach, and like-group cohesion has an independent effect on the outcome [24].

The group moderator plays an important role in facilitating such positive group processes and hence outcomes [11,13,14,25,26]. Often, this moderator is a trained psychologist, therapist, or other professional with relevant skills and knowledge to facilitate the group. Facilitation methods that moderators can use include role modeling, psychological education, setting rules of communication by appointing turns, framing, supporting, and initiating themes and activities, with the goals of creating psychological safety and respectful interaction while enabling participants to feel free to share ideas and concerns [27]. Group moderators can emphasize member interaction, create a positive group climate, and handle conflicts immediately upon occurrence to help develop and maintain group cohesion and therapeutic alliance with group members [11,16,17].

### Group Characteristics

For group characteristics, the literature points to the relevance of homogeneity, a certain group size, and setting and environment. To start with, homogeneity refers to similarities between group members, which can include age, cultural identity, or, for instance, similar health problems. Group homogeneity has been found to enhance both group cohesion and group identification by establishing a sense of being equal [5,10,28,29] and may reduce experiences of social stigma and lower the threshold to share sensitive information [5,10]. A meta-analytic review by Burlingame and colleagues [30] revealed that groups that were more homogeneous improved more compared to their heterogeneous counterparts.

Next, although there is no particular evidence on the most effective group size, it is generally recommended to hold groups with between 8 to 12 people [6,13,31]. The rationale for this is that while too-small groups may hamper interaction and exchange, too-large groups may undermine the interaction between group members [6].

Furthermore, setting and environment play an essential role in group interventions in general [25]. According to Weinberg [25], managing the setting in which group therapy takes place is an essential element to consider. “Creating a holding environment” may involve a certain choice of furniture, seating order, and placement of a box of tissues for participants. Additionally, calming music in certain areas, such as waiting rooms, may create the impression that the therapist or moderator is taking care of the participants’ needs.

Generally, small health-related group sessions take place in a face-to-face fashion, where people interact in a group in a particular setting. However, since the COVID-19 outbreak, face-to-face group meetings have no longer been able to proceed in their original form. Whereas in many cases these group interventions were postponed, as it was believed that the essential requirements for small group processes could not be met, people also started experimenting with online group meetings, shifting to a digital environment [25,32]. It is likely that these kinds of online interventions remain.

As was shown in a meta-analysis of studies of the effectiveness of internet-based interventions for therapy by Barak and colleagues [7], outcomes of online and face-to-face groups are comparable in terms of effectiveness (eg, [10,25,32,33]). These findings are related to group intervention studies in the context of physical activity–related behavior [14], treatment of anxiety disorders [34,35] or depression [35,36], and the promotion of personal empowerment in online support groups for patients with dental anxiety [7]. These results lead to the conclusion that online and face-to-face groups have comparable effectiveness in different health care contexts and in domains varying from psychosocial effects to treatment outcomes (ie, lowering depressive symptoms) [37]. Yet looking back at crucial group elements, such as homogeneity, group setting and environment, and the establishment of supportive relationships between group members (ie, group cohesion) and with the moderator (ie, therapeutic alliance), shows that the online setting may provide both challenges and opportunities with regard to these group characteristics and group processes that are less well-known.

In this paper, we therefore aim to explore (1) potential challenges that may arise when providing health-related interventions to groups in an online setting, (2) how these challenges can be overcome or avoided, and (3) what possible advantages arise from the online format. We reviewed the literature to address these questions, with a focus on synchronous groups, that is, those in which individuals come together online with the aim to participate in any sort of group activity at the same time, such as to learn, share experiences, change health-related behaviors, and support one another via screen teleconferencing [26]. These synchronous, online, group-based health-related programs are often led by a trained peer or a professional (eg, a psychologist, therapist, or other relevant professional).

## Methods

### Overview

In this study, we conducted a scoping review; that is, an exploration of a topic that is less well-established in the literature to provide a first overview and potential requirements. In our review, we aimed for a general up-to-date overview of various publications to allow for a comprehensive outline instead of answering a more narrow or specific research question. Where applicable, PRISMA (Preferred Reporting Items for Systematic Reviews and Meta-Analyses Extension for Scoping Reviews) guidelines were followed.

### Eligibility Criteria

We included published reports and studies that (1) concerned adult populations taking part in a face-to-face or synchronous online group or support group (this included group therapy and group interventions, thereby excluding educational small group meetings in organizational team-building contexts and online forums or chat groups where individuals do not necessarily come together to engage at the same time), (2) were conducted in a psychological, clinical, or health promotional setting and were led by a moderator (often a psychologist or other professional with relevant expertise), (3) explored effectiveness or provided definitions of group interventions, and (4) reported behavioral outcomes relevant to healthy eating, physical activity, mental health (eg, quality of life), smoking cessation, or health status in any way. Studies published from 2000 to 2021 were eligible. We chose to include studies published within the last 2 decades to ensure relevance to current social, health, and health care climates.

### Information Sources

The search took place from February 2021 to June 2021 and was carried out on Scopus and Google Scholar.

### Search Strategy

Search terms referred to relevant constructs and included *small groups*, *group intervention*, *support groups*, *online group therapy*, *videoconferencing*, *online*, *face-to-face*, and *web-based*. Furthermore, we included essential core elements in our search, such as *group cohesion*, *therapeutic alliance*, and *essential elements of groups*. Additional articles were further snowballed via relevant articles until saturation of results relating to the research questions was reached.

## Results

The search resulted in a large number of relevant publications that could answer the 3 research questions central to this study, including meta-analyses (eg, [7,17]), systematic reviews (eg, [8,20,32,38]), effect studies (eg, [3,35]), publications providing theoretical frameworks and informing the design of online groups (eg, [1,2,10,39,40]), and articles elaborating on past experiences of relevant stakeholders in the context of online groups (eg, [9,25,41]). Relevant research reports were in the contexts of health promotion and disease prevention relating to group support or therapy (eg, [7,25,30]), group processes and dynamics (eg, [1,5]), and specific characteristics of group interventions (eg, [6,10]). In our review, we extracted relevant information from these articles that seemed to partly overlap in outcomes. We recognize that this approach is not reproducible and bears the potential of incompleteness.

### Challenges

The identified publications pointed to several challenges that need to be considered when providing small group interventions in an online setting. To start with, in light of the importance of building up group cohesion, one major challenge seems to be the lack of nonverbal communication in an online setting. The absence of body-to-body interaction, with the absence of eye contact being especially relevant and fundamental for group therapy, can be seen as one of the main obstacles in an online setting [9,25,42]. This is due to the fact that an online setting makes it difficult to read and react to both body language and nonverbal signs. Additionally, an online setting hinders affect regulation by the moderator and may make it difficult to express desired messages clearly [41]. The conveyance of nonverbal cues during therapeutic communication is one important part of displaying empathy and affect, reassuring and boosting disclosure, evolving alliance, authenticating care, and ensuring authenticity, among other functions [9]. Recent research implies that online group members do not feel as connected to other group members as in-person group members, which is indicative of lower group cohesion [43].

Another challenge to be tackled is the establishment of a therapeutic alliance [44]. In their review, Gentry et al [32] examined the extent to which therapeutic alliance can be maintained in online group-based treatment. They found that the online setting may result in small decreases in therapeutic alliance.

Some challenges pertain to the setting of the group. In face-to-face groups, the moderator normally controls the setting in which the sessions take place. This includes the arrangement of seats and the environment, the placement of boxes of tissues or plants, and even the choice of music to promote a calm and welcoming experience. These functions, also referred to as “dynamic administration” [45], include the overall setup of the group and handling of the time and space of the meeting, as well as matters concerning boundaries. Since the environment in the online setting is partly dependent on the participants themselves, the moderator requires the participants to prepare a “holding” environment for themselves, such as a quiet place where they feel free to open up and speak.

The online setting also needs to be accounted for, as it offers potential distractions that may not play a role in a face-to-face environment [25]. These include background noises and individuals outside the group setting who are in or enter the same space as the participant and distractions due to the chosen platform [25].

Aside from the elements essential for face-to-face group interventions, online group interventions need to handle technical concerns. More specifically, technical problems such as consistency and speed of the internet connection and overall technical infrastructure, including audio or visual difficulties, delays, dropout, background noise, and poor lighting, may be a limitation [9,25,42]. While offering increased access and recruitment for certain groups, these technical issues can potentially lead to the exclusion of participants who do not have access to a computer, technology, or the internet [9,25,39]. Older participants who are less tech-savvy may therefore be especially at risk of exclusion or be less able to easily access the group [25,39]. The same holds true for individuals who due to their condition may not be able to sit behind a screen for a long period of time [10,39]. Additionally, online groups may be less suitable for participants who are prone to or are currently in acute distress or easily deregulated (such as severely depressed participants or those with suicidal ideation) [9,25]. Reaching out to the aforementioned participants when intervention is needed can be difficult or even impossible, as doing so usually requires more time and attention than the group can provide, especially when it is conducted online [9].

### How to Overcome Challenges

Some reports in the literature suggest that participants in online groups experience less group cohesion than face-to-face group participants ([43], see above). Other reports [9,46] suggest that to build up group cohesion and reduce the effect of the absence of bodily interaction and nonverbal cues, moderators can stimulate the presence and input of all participants by directing them to provide input, verbalize what they take from others’ contributions, check in on how they feel, and actively identify mutual understanding (eg, “I see many of you nodding, so it seems like you agree with what Jennifer said”). In order to guarantee adequate and effective metacommunication, the literature [25,29,46] recommends that group moderators consider receiving skills training beforehand. Metacommunication can be defined as “communication about communication” [47], which in the case of the online setting relates to participants verbalizing thoughts and feelings that are evoked by what others say; other participants might otherwise miss these due to the lack of eye contact and body language.

Furthermore, we found that moderators should ensure that time is given to every participant by distributing turns, and that they should acknowledge feelings by verbalizing observations [9,46]. Although group cohesion among online groups might be less strong than in face-to-face groups, the overall convenience of online group sessions seems to outweigh the negative factors of the online setting [43].

Establishment of therapeutic alliance was found to be a second challenge. Based on a literature review and practical experiences, Kneeland et al [46] described five strategies to facilitate therapeutic alliance in a group-based videoconferencing setting: (1) explicitly express gratitude to group members, (2) start the group with an introduction exercise with all attendees and use “ice breaker” questions, (3) self-disclose the group moderator to humanize the face on the screen and build rapport, (4) provide validation, which is the recognition of someone’s feelings and thoughts to underscore that listening is nonjudgmental, and (5) promote rapport between group members and the moderator [48].

As with face-to-face groups, online groups can establish ground rules. These may relate to respectful communication concerning the online setting, such as the use of the camera and microphone during sessions, as well as how to transparently deal with events such as other people entering a participant’s home environment [9,25,29]. To facilitate an online session, a moderator may coordinate activities such as breakout rooms. To ensure the provision of an overall positive experience, Lalande et al [29] recommend that group moderators obtain feedback from group participants during, as well as after, the session.

The literature suggests that is the responsibility of the moderator to consider a digital format that is easily accessible, safe, and convenient for participants to use [25,29]. This comprises the use of an online consent form and making sure the online platform is indeed accessible to users [29].

To overcome technical challenges and ensure that the chosen platform is safe, moderators should run a pilot in order to test whether the session can go through as planned [29,46]. According to Lalande et al [29], participants may benefit from instructions on how to log in and navigate through the digital platform, including tips in case they encounter any problems. These instructions should be offered to participants prior to the start of the session. Furthermore, in order to successfully start the session, the literature advises moderators to make sure that all participants can log in before the session starts and to invite feedback on technological aspects throughout the session [29].

As moderators in online settings are often unable to intervene with participants who require an intervention during imminent emergency situations, Stephen et al [9] recommend that participants be asked to provide contact details (eg, their whereabouts or address and the phone number of an emergency contact) in case of emergency.

### Opportunities

While there are certain challenges to overcome in online group settings, there are also opportunities. One of the opportunities of online groups as opposed to face-to-face groups is group size. Commonly used online platforms, such as Zoom or Microsoft Teams, allow for groups of up to roughly 50 participants to be seen on the screen at once. As it remains to be discussed if such a high number of participants is desirable, limiting online group intervention sessions to 15 participants (as suggested in the literature) therefore seems preferable. However, communication in an online setting may require more effort and moderating strategies, which will be discussed later on.

Furthermore, the online setting provides an opportunity to manipulate group composition due to increased accessibility for individuals who face challenges meeting in person [10,29]. For example, online settings are accessible to people with rare diseases or disabilities, in certain sociodemographic groups, or who are otherwise excluded for reasons such as transportation difficulties, distance, mobility problems, or caregiving responsibilities [7,9,10,39,42,43]. The online setting offers individuals the chance to connect even across the globe, including individuals living in rural areas, thereby enriching demographic diversity while promoting homogeneity of the group [29,43]. Moreover, the possibility to access group interventions online can be time-saving, cost-effective, and convenient due to decreased travel costs and time demands on participants, as they can participate from the comfort of their home [25,39].

While shared characteristics of participants can promote a sense of safety due to decreased stigma and felt recognition, the screen barrier separating participants from each other may be seen as another opportunity, as it may stimulate that feeling even more. The anonymity that can more easily be realized in online groups seems to reduce stigmatization [7,10,25,39,49], power differentials (through neutralizing of status) [9,39], and, consequently, potential inhibition of participants who may otherwise not dare to speak up [9]. As an additional benefit, taboos can be discussed more freely and participants can be encouraged to self-disclose [7,39]. Participants may thus perceive less rejection, which in return promotes honest discussions of feelings and otherwise avoided topics. This seems especially true for male individuals, who have been found to participate more freely in online settings, notably when sensitive topics are discussed, such as suicide and depression [39]. At the same time, group interventions can at times be emotionally overwhelming for participants, which is why the screen barrier may lead to less negative mental impact and defensiveness, as participants may feel sheltered behind their screens [7,9,25]. Socially anxious participants and participants with dissociative symptoms may especially gain from this approach, as they may experience less anxiety and lower their dissociative defenses more easily due to reductions in immediacy and a sense of self-consciousness [25]. Participants with a borderline personality disorder diagnosis may also benefit from the screen barrier due to a greater distance from the therapist, leading to the perception of online groups as being safer [25].

## Discussion

### Principal Results

In this paper, we elaborate on potential challenges when executing small, synchronous group interventions in an online setting, how these challenges can be overcome or prevented, and what possible opportunities arise from the online format. Essential factors related to small groups include group processes, such as group cohesion, therapeutic alliance, self-disclosure, and feedback, as well as factors relating to the characteristics of the group, such as the size and composition of the group and its setting and environment. From our review of the literature, we conclude that while comparable group outcomes and group processes can be realized in online settings and in offline, face-to-face group settings, both may come with specific benefits and challenges that need to be addressed. On the one hand, challenges include the lack of nonverbal communication, which impacts the establishment of a therapeutic alliance and group cohesion; potential technical concerns; and a lack of suitability for certain participant groups, such as those in acute crisis. Yet the literature suggests measures and strategies to avoid or overcome these pitfalls. Some can be overcome by moderators improving their communication skills (eg, by practicing metacommunication, such as disclosing their own feelings, distributing turns, and recognizing participants’ feelings), technical measures (eg, choosing a secure platform, running a pilot test, and providing participants with instructions), and setting the environment (eg, establishing ground rules). While most of the effort to make online group sessions work falls on the moderator, participants themselves can play an active role by ensuring they take the time and make the effort to prepare a holding environment for themselves and the other participants, give feedback to the moderator, and provide the moderator with relevant contact details and their whereabouts in case of an emergency situation.

On the other hand, we encountered some advantages of providing group interventions in an online setting. The advantages include convenience (eg, saving time and being cost-effective), accessibility, and inclusion; the online setting enables individuals to connect with each other who may not otherwise have come together in a face-to-face setting. Furthermore, the screen barrier and higher perception of anonymity may promote participants’ sense of safety, potentially leading to a decrease in stigmatization of topics and self-inhibition, thereby encouraging self-disclosure.

Previously, scholars [50,51] have stressed that essential conditions of behavior change methods (eg, stimulating group cohesion) need to be met when translating these methods into practical applications. We hope we have provided some guidelines to intervention designers and practitioners on how the essential conditions of small group interventions can be created in an online context.

### Strengths and Limitations

This review was not carried out in a systematic way, nor does it offer a quantitative overview of the effectiveness of online groups. While this means that the reproducibility of the review is low and its completeness cannot be guaranteed, it can offer an up-to-date scoping overview of current knowledge and relevant considerations when transferring or organizing small group interventions in an online setting. Given contemporary developments in overall digitization and changing regulations concerning, among other topics, the COVID-19 pandemic, this discussion seems to be eminently relevant.

### Conclusions

The COVID-19 pandemic has emphasized the need to continue group interventions while switching to an online setting [25]. Even though face-to-face groups are starting to return, online groups seem to be the “new normal” in many cases, implying the possibility that more and more group interventions may transfer to an online setting. While online groups offer a considerable number of new possibilities and benefits compared to face-to-face groups (eg, accessibility, the screen barrier, and time effectiveness), there are pitfalls to consider and avoid when setting up an online group (eg, technical concerns and ensuring that emergency contact details are available for participants), as executing online group interventions demands thorough preparation and, in some cases, even extra training in order to maximize effective group outcomes. This includes actions executed before, during, and after the group sessions that relate to group characteristics in terms of the frame and overall setting, technical aspects of the sessions, and group moderation, as well as attention to group processes and participant care. In conclusion, online groups may be a very suitable way to support individuals in groups, not only when face-to-face meetings are difficult or impossible, but even under normal circumstances, given the numerous benefits and possibilities of the online setting.

## Abbreviations

PRISMA: Preferred Reporting Items for Systematic Reviews and Meta-Analyses Extension for Scoping Reviews
